# Chemical Evaluation and Nutritional Benefits of Dietary Additives Formulated From Fruit Peel Blends

**DOI:** 10.1002/fsn3.70414

**Published:** 2025-06-22

**Authors:** Eridiong Onyenweaku, Hema Kesa, Patricia Ebai

**Affiliations:** ^1^ Food Evolution Research Laboratory, School of Tourism & Hospitality College of Business & Economics, University of Johannesburg Johannesburg South Africa; ^2^ Human Nutrition & Dietetics Faculty of Allied Health Sciences, University of Calabar Calabar Nigeria; ^3^ Higher Teacher Training College for Technical Education, University of Duoala Duoala Cameroon

**Keywords:** antioxidant, blends, dietary additives, food waste, fruit peels, nutrients

## Abstract

Food insecurity and malnutrition remain global concerns, exacerbated by the wastage of nutrient‐rich plant foods and their underutilized parts. Fruit peels, often discarded in food consumption and processing, contribute to substantial nutrient loss and food waste. This study formulated and evaluated different fruit peel blends and assessed their micronutrient composition, phytochemical content, and antioxidant activity. An experimental approach was employed, processing nine fruit peels into three primary blends: orange‐lemon‐mandarin (OLM), pineapple‐cucumber‐green apple (PCG), and watermelon‐guava‐red apple (WGR), each with three variations (A, B, C). The fruit peels were cleaned, dried, de‐bittered, and ground into powder before laboratory analysis using AOAC standard methods. Data were statistically analyzed using analysis of variance (ANOVA) on Statistical Package for Social Science (SPSS) version 20.0, with significance accepted at *p* < 0.05. The results revealed significant variations in micronutrient concentrations and antioxidant activity. Vitamin A content ranged from 9.41 ± 0.01 mg/100 g (WGRC) to 28.8 ± 0.06 mg/100 g (PCGC). Among the vitamins, ascorbic acid was the most predominant, with PCGB recording the highest value (45.3 ± 0.02 mg/100 g). Flavonoid content varied significantly, from 42.76 ± 0.01 mg/100 g (PCGC) to 76.83 ± 0.04 mg/100 g (OLMB). Phytate and oxalate were also present, with OLMA having the highest total phenol content (19.80% ± 0.01%). PCGC exhibited remarkable antioxidant activity, being highest in carotenoid, Ferric Reducing Antioxidant Power (FRAP), and metal chelating activity. The blends also contained essential micro and macro elements, with OLMA recording up to 2.2 ± 0.03 mg/100 g of iron. These findings highlight the nutritional potential of fruit peels, advocating for their use as dietary additives to reduce waste and enhance nutritional value. Promoting their processing and consumption could contribute to sustainable nutrition solutions.

## Graphical abstract

Fruit peels, often discarded in food consumption and processing, contribute to substantial nutrient loss and food waste. This study formulated and evaluated different fruit peel blends and assessed their micronutrient composition, phytochemical content, and antioxidant activity. The findings highlight the nutritional potential of fruit peels, advocating for their utilization as dietary additives to reduce waste and enhance nutritional value.

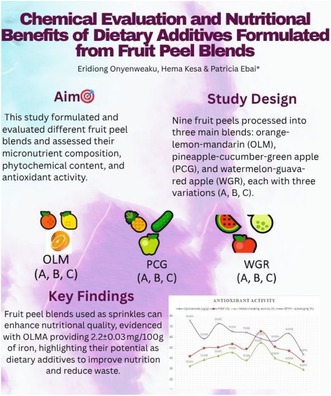

## 1 Background

The emphasis on disease prevention and health enhancement is increasing, aligning with the Millennium Development Goal of achieving sustainable health and well‐being. Functional foods and nutraceuticals, which provide health benefits beyond basic nutrition, are gaining widespread recognition and popularity (Rajendran et al. 2014; Ashraf et al. 2024). Scientific studies have demonstrated the role of healthy diets in preventing both chronic and infectious diseases. In recent times, there has been a mindful shift from animal foods to the promotion of plant‐based foods for the prevention and management of diet‐related diseases which have been on the rise (WHO 2014).

Fruit peels, often discarded as waste, contain valuable nutrients and bioactive compounds that could be repurposed to address malnutrition and sustainability issues (Schieber et al. 2001; Feumba et al. (2016). On the other hand, food waste, particularly fruit peels, is a significant global challenge contributing to environmental issues. However, fruit peels are rich in phytochemicals, dietary fiber, vitamins, and minerals (Feumba et al. 2016; Onyenweaku and Kesa 2024). Global food waste is estimated at 1.3 billion tons annually, with fruit and vegetable waste forming a significant portion; but beyond the environmental impacts, food wastage costs some $750 billion annually to food producers (UN 2013). Enhancing the nutritional potential of food systems by incorporating underutilized resources like fruit peels can help address both malnutrition and food insecurity (Hunter et al. 2019).

Furthermore, apart from enhancing the nutritional value of meals these fruit peels are added to, developing products from them aligns with global sustainability goals (UN SDG 12: Responsible Consumption and Production). Feumba et al. (2016) report that utilizing fruit by‐products, particularly peels, which can represent up to 30% of the total weight in some fruits, has gained momentum, especially as research has revealed their superior biochemical activities compared to other fruit parts. Moreover, with the growing interest in natural sources of bioactive compounds and the popularity of functional foods these days, the development of food products enriched with fruit peels is being promoted (Babiker et al. 2013; Altunkaya et al. 2013). Similarly, individuals can also simply process fruit peels to be added to their meals as sprinkles. Farmers can also be incentivized to utilize fruit peels that are typically discarded during juice production and other fruit processing activities. By processing and packaging these peels for commercial sale, they can contribute to waste reduction while simultaneously creating an additional revenue stream. This approach promotes both environmental sustainability and economic empowerment within the agricultural sector.

On the other hand, the world is also battling with the challenge of child malnutrition and micronutrient deficiencies (also known as hidden hunger); both remain significant global public health challenges, particularly in low‐ and middle‐income countries. Nearly half of deaths among children under 5 years of age are linked to undernutrition. These mostly occur in low‐ and middle‐income countries. The developmental, economic, social, and medical impacts of the global burden of malnutrition are serious and lasting for individuals, families, communities, and countries (WHO 2024). The conventional approach to combating malnutrition relies heavily on staple crops, food fortification, and supplementation programs. However, an alternative and sustainable approach is the use of nutritious underutilized plants, as well as uncommonly consumed plant parts such as medicinal seeds, peels, and bark, which can provide essential micronutrients, improve dietary diversity, and protect against diseases.

Many underutilized plants are reported to have superior nutritional profiles compared to conventional staple crops. For example, Baobab ( *Adansonia digitata* ) fruit pulp is an excellent source of vitamin C, which enhances iron absorption and boosts immunity (Chadare et al. 2018). In the same vein, many researches like that of Low et al. (2007) have reported orange‐fleshed sweet potato ( *Ipomoea batatas* ) to be rich in beta‐carotene, a precursor of vitamin A, which is crucial in preventing childhood blindness and improving immune health. Hidden hunger, characterized by deficiencies in essential micronutrients such as iron, zinc, iodine, and vitamin A, affects millions of children worldwide, leading to stunted growth, impaired cognitive development, and weakened immunity (Lowe 2021). Integrating underutilized plants and uncommonly consumed parts into children's diets can go a long way in addressing these micronutrient deficiencies.

Despite their potential, underutilized plants/plant parts face challenges such as low consumer awareness, limited market access, and inadequate research on their agronomic and nutritional properties. In order to maximize their benefits, in‐depth research to evaluate the chemical composition of plant food materials is necessary. This will provide information on the potential uses of certain plants/plant parts in the prevention, treatment and management of illnesses, followed by awareness campaigns to enhance their acceptance and consumption. Consequently, this study formulated and assessed the micronutrient, antioxidant activity, and phytochemical content of nine fruit peel blends, which can be used as dietary additives.

## 2 Materials and Methods

This research design was experimental, employing a quantitative approach.

### 2.1 Study Area

This research was carried out in Calabar, Cross River State in Southern Nigeria. Most of the samples were purchased from the local markets and a few from local farmers in the area.

### 2.2 Sample Collection and Preparation

The fruits were purchased fresh and whole; about 3 kg each of matured samples of orange ( *Citrus sinensis* ), lemon ( *Citrus limon* ), mandarins ( *Citrus reticulata* ), pineapple ( *Ananas comosus* ), cucumber ( *Cucumis sativus* ), green apple (*Malus domestica—golden*), watermelon ( *Citrullus lanatus* ), guava ( *Psidium guajava* ), and exotic red apple (*Malus domestica—red delicious*) fruits were collected from local markets and farmers. To ensure accurate results, special care was taken to select fruits that were free from visible blemishes and bruises. Ripe fruits were purchased for this study and used immediately for analysis.

### 2.3 Sample Preparation

**FIGURE 1:**
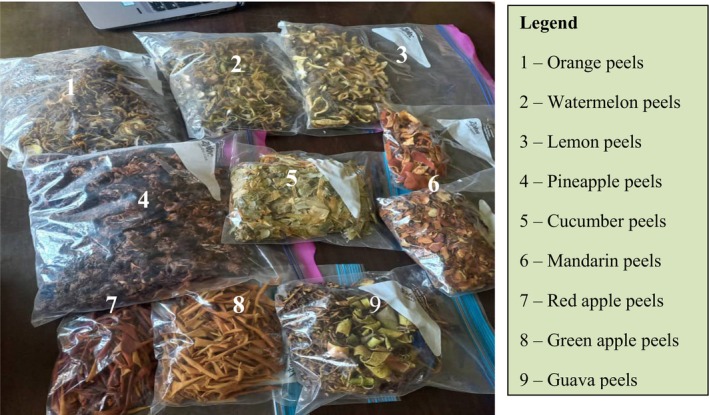
Some of the dried fruit peels used to formulate the blends.

Prior to analysis, the fruits were prepared by first sorting, then washing them thoroughly under running water to remove any surface dirt, debris, or other contaminants before draining. The fruits were then peeled carefully using either a scraper or a sharpened knife to avoid the inclusion of fruit pulp or albedo, which is an inner layer of spongy white tissue in citrus fruits. Finally, the fruit peels were oven‐dried at 50°C to remove excess moisture, then placed in air‐tight, well‐labeled Ziploc bags. The process of de‐bittering was carried out on the dried fruit peels in order to remove bitter principles from the peels (Figure 1).

#### 2.3.1 De‐Bittering

The de‐bittering process was carried out using 2 g/100 mL of sodium bicarbonate (NaHCO_3_). The samples were soaked in the NaHCO_3_ at a ratio of 1:50. The soaked samples were left for 45 min after which they were boiled for 1 h. The samples were allowed to cool and then filtered before drying them at 80°C. This was done according to the method described by Manjarres‐Pinzon et al. (2013). After this, the dried, de‐bittered peels were dried, then ground to obtain a fine powder using a Binatone (BLG 620) blender at 3000 rpm. These steps were taken to ensure that the samples were in a consistent state for chemical analysis and to minimize any potential variability in the results due to differences in moisture content or other factors. The fruit peels were then weighed and formulated into blends as shown in Table 1 putting into consideration the widespread availability and popularity of those peels in that region of the country.

**TABLE 1:** Formulation of the fruit peel blends.

Blends	% by weight of fruit peels		
CITRUS – OLM	Orange	Lemon	Mandarin
OLMA	80	10	10
OLMB	70	20	10
OLMC	60	30	10

PCG	Pineapple	Cucumber	Green apple
PCGA	80	10	10
PCGB	70	20	10
PCGC	60	30	10

WGR	Watermelon	Guava	Red apple
WGRA	80	10	10
WGRB	70	20	10
WGRC	60	30	10

### 2.4 Vitamin Analysis of the Fruit Peel Blends (Detailed Methodology in Appendix)

#### 2.4.1 Determination of Vitamin A Content

The AOAC (2010) colorimetric method was used to measure vitamin A by detecting its reaction with SbL_3_ at 620 nm (Appendices 1 and 2).

#### 2.4.2 Determination of Vitamin B_1_ Content

Thiamine (B_1_) content was determined using the scalar analyzer method of AOAC (2010).

#### 2.4.3 Determination of Vitamin B_2_ Content

Riboflavin (B_2_) was determined according to AOAC (2010) fluorimetric method.

#### 2.4.4 Determination of Vitamin B_3_ Content

The spectrophotometric method described by Okwu and Ndu (2006) was employed for niacin (B_3_).

#### 2.4.5 Determination of Vitamin C Content

Ascorbic acid was determined using the titrimetric method described by AOAC (2010).

#### 2.4.6 Determination of Vitamin E Content

Vitamin E was determined using the method described by AOAC (2010).

### 2.5 Mineral Analysis of the Fruit Peel Blends

#### 2.5.1 Determination of Macroelements

This was carried out by elemental assay using Atomic Absorption spectrophotometer (Perkin Elmer Model 306, UK) as described by Nnadi (2020). Standard for each element was run, and the sample digest was aspirated. The content of the element was calculated using the formula below:

$$\text{Metal}\;\left(\%\right)=\frac{C\times V}{10^{4}\times \mathrm{SW}}$$

where, *C* = concentration, ppm; *V* = solution volumes, SW = sample weight.

##### 2.5.1.1 Determination of Magnesium

The AAS was set up using a magnesium hollow cathode lamp at a wavelength of 202.6 nm. Magnesium standard solution of the following concentrations of 0.5, 1.0, 2.0, and 4.0 ppm was prepared from the standard stock solution. The equipment was zeroed using de‐ionized water. Magnesium standard was run to obtain the standard plot. The sample digest was run, and the values displayed on the screen were also recorded; magnesium concentration was calculated using the formula above.

##### 2.5.1.2 Determination of Potassium

The Atomic Absorption spectrophotometer was stabilized after mounting the potassium hollow cathode lamp and setting the wavelength to 766.5 nm. Potassium standards (5, 10, 15, and 20 ppm) were prepared from stock solutions and used for calibration to generate a standard curve. The sample digest was analyzed, and the displayed values were recorded. The potassium content was then calculated using the specified formula.

##### 2.5.1.3 Determination of Sodium

The concentration of sodium was analyzed using a sample digest prepared by digesting completely 5 g of the sample in perchloric and concentrated nitric acids diluted with deionized water in a 50 mL volumetric flask. The sample digest was run and the values on the screen displayed was recorded. The sodium content was calculated using the formula as provided.

##### 2.5.1.4 Determination of Calcium

The AAS was configured with a calcium hollow cathode lamp at a wavelength of 422.7 nm. Calcium standards (0.5, 1.0, 2.0, and 3.0 ppm) were prepared from a standard solution and used for calibration to generate a standard curve. The sample digest was analyzed, and the displayed ppm values were recorded. The calcium content was then calculated using the provided formula.

#### 2.5.2 Determination of Microelements

The microelement content of the samples was analyzed using the Atomic Absorption Spectrophotometric method (AOAC 2020). Samples were digested with a nitric, perchloric, and sulfuric acid mixture, then filtered and diluted to 50 mL with distilled water. Standard solutions (1000 ppm in 2N nitric acid) were prepared, and calibration and measurement were conducted at specific wavelengths (as seen in Table 2) against a blank.

**TABLE 2:** Elements and their AAS wavelength.

S/No	Elements	Wavelength (nm)
1	Zinc	213.8
2	Iron	234
3	Manganese	246
4	Selenium	384
5	Copper	206

$$\text{Calculation}:\text{Element}=\frac{A_{u}}{A_{s}}\times \mathrm{ppm}\;\text{of curve}\times \frac{V_{f}}{A_{s}}\times \frac{1000}{W}$$

*A*_u_ = absorbance of sample, *A*_s_ = absorbance of standard, *V*_f_ = total volume of extract, *V*_x_ = total volume of extract used, *W* = weight of sample used.

### 2.6 Antioxidant Activity

#### 2.6.1 Analysis of Carotenoids

Total carotenoid content was determined using the acetone extraction method (Rodriguez‐Amaya and Kimura 2005). A 5 g sample was ground with 3 g of hyflo super‐cel in 50 mL of cold acetone, filtered, and mixed with 40 mL of petroleum ether in a separating funnel. Distilled water (300 mL) was added, and the lower phase was discarded. The extract was washed with distilled water, passed through glass wool and anhydrous sodium sulfate, then absorbance was measured at 450 nm using a UV–visible spectrophotometer.

#### 2.6.2 Determination of 1,1‐Diphenyl 1.2‐Diphenyl 2‐Picrylhydrazyl Free Radical‐Scavenging Ability (DPPH)

The antioxidant activity of the extracts was measured in terms of hydrogen‐donating or radical‐scavenging ability using the stable free radical (DPPH) was evaluated as described by Afify et al. (2012). A 50 μL sample and 450 μL Tris–HCl buffer (pH 7.4) were mixed, followed by 1.0 mL DPPH‐methanol solution. After 30 min in the dark, absorbance was measured at 517 nm, and inhibition percentage was calculated.

$$\text{Inhibition}\%=\left[\left({\mathrm{Abs}}_{\text{ctrl}}-{\mathrm{Abs}}_{\text{sample}}\right)/{\mathrm{Abs}}_{\text{ctrl}}\right]\times 100\%$$

where: Abs_ctrl_ is the absorbance of a control and Abs_sample_ is the absorbance of a sample.

#### 2.6.3 Determination of Ferric Reducing Antioxidant Power (FRAP)

The reducing power of each sample was determined using the FeCl_3_ reduction method (Oyaizu 1986). The extract was mixed with potassium ferricyanide, incubated at 50°C for 20 min, and treated with trichloroacetic acid. After centrifugation, the supernatant was combined with FeCl_3_ , and absorbance was measured at 700 nm. Higher absorbance indicated greater reducing power, and antioxidant capacity was calculated.

#### 2.6.4 Determination of Metal Chelating Activity

Iron (II) chelation assay was performed according to the method used by Chew et al. (2009). A 0.675 mL sample and 0.075 mL Iron (II) solution were mixed and reacted for 20 min. Ferrozine solution (0.75 mL) was added, vortexed, and left for 5 min. Absorbance was measured at 562 nm. EDTA standards (0–100 μM) were used for calibration. Samples were tested at 0.5 and 1 mg/mL in five replicates.

### 2.7 Phytochemical Analyses (Please See Appendix for the Full Methodology)

#### 2.7.1 Determination of Tannin, Phytate and Hydrogen Cyanide (HCN) Content

For these three, the folin‐denis spectrophotometric method as described by AOAC (2005) was used (Appendices 1 and 2).

#### 2.7.2 Determination of Oxalate

Oxalate was determined titrimetrically using the method described by Falade et al. (2004), Munro and Bassir (1969).

#### 2.7.3 Determination of Flavonoid and Total Phenolic Content (TPC)

The total flavonoid and phenolic content was determined according to the method of Meda et al. (2005) using gallic acid as standard.

### 2.8 Statistical Analysis

Laboratory results generated from this study were statistically analyzed using the Statistical Package for Social Science (SPSS) version 20.0. One‐way analysis of variance (ANOVA) was used to compare the nutrient values and check for significant differences across the samples. All the results were expressed as mean ± SEM for 3 determinations, and statistical significance was accepted at *p* < 0.05 (95% confidence level). SPSS version 20.0 was used to carry out ANOVA.

## 3 Results

### 3.1 Vitamin Composition of the Fruit Peel Blends

Table 3 presents the vitamin contents of the nine fruit peel blends in this study. The values are expressed as mean ± SEM, with different superscripts in the same column indicating significant differences (*p* < 0.05) between the samples. There were notable variations in the individual vitamin content among the nine samples. The β‐carotene (Vitamin A precursor) levels varied significantly across the samples, with PCGC (28.8 ± 0.06 mg/100 g) and PCGB (28.4 ± 0.04 mg/100 g) exhibiting the highest concentrations, which were significantly higher than those found in others; OLMB had the least β‐carotene (5.4 ± 0.01 mg/100 g). Vitamin B_1_ did not vary much among the samples, with values ranging just between 0.4 ± 0.01 and 0.5 ± 0.04 mg/100 g. A similar trend was observed for vitamins B_2_, B_3_, and E values, with a few blends recording concentrations that were significantly different from others. On the other hand, Vitamin C content varied significantly (*p* < 0.05) across the nine samples, with PCGB recording the highest value (45.3 ± 0.02 mg/100 g), while WGRC had the lowest content (21.4 ± 0.03 mg/100 g). Ascorbic acid was the most predominant vitamin in the samples analyzed, followed by β‐carotene.

**TABLE 3:** Vitamin content of the fruit peel blends in mg/100 g.

Samples	β‐carotene	Vitamin B_1_	Vitamin B_2_	Vitamin B_3_	Vitamin C	Vitamin E
OLMA	9.9 ± 0.01^c^	0.4 ± 0.01^b^	1.0 ± 0.01^b^	1.0 ± 0.01^a^	32.4 ± 0.02^f^	1.9 ± 0.03^b^
OLMB	5.4 ± 0.01^a^	0.5 ± 0.01^c^	1.2 ± 0.04^d^	1.2 ± 0.04^c^	25.8 ± 0.03^b^	2.0 ± 0.01^c^
OLMC	18.4 ± 0.02^d^	0.5 ± 0.01^c^	1.1 ± 0.04^c^	1.1 ± 0.04^b^	34.8 ± 0.01^g^	2.2 ± 0.03^d^
PCGA	22.4 ± 0.01^g^	0.4 ± 0.02^b^	1.2 ± 0.03^d^	1.2 ± 0.03^c^	25.8 ± 0.06^c^	1.9 ± 0.01^b^
PCGB	28.4 ± 0.04^h^	0.4 ± 0.01^b^	1.0 ± 0.01^b^	1.1 ± 0.01^b^	45.3 ± 0.02^i^	2.2 ± 0.03^d^
PCGC	28.8 ± 0.06^i^	0.4 ± 0.01^b^	1.2 ± 0.05^d^	1.2 ± 0.04^c^	26.4 ± 0.03^d^	2.6 ± 0.04^e^
WGRA	13.1 ± 0.01^e^	0.5 ± 0.04^c^	1.1 ± 0.01^c^	1.1 ± 0.01^b^	36.4 ± 0.03^h^	2.2 ± 0.04^d^
WGRB	10.2 ± 0.01^d^	0.4 ± 0.01^b^	1.0 ± 0.01^b^	0.9 ± 0.01^a^	28.4 ± 0.04^e^	1.5 ± 0.04^a^
WGRC	9.4 ± 0.01^b^	0.3 ± 0.01^a^	0.6 ± 0.01^a^	1.0 ± 0.01^a^	21.4 ± 0.03^a^	2.8 ± 0.01^f^

*Note:* Values in the same column with different superscripts are significantly different at *p* < 0.05. Values are expressed as mean ± SEM, *n* = 2.

### 3.2 Mineral Concentrations of the Fruit Peel Blends

#### 3.2.1 Macro Elements

The macro element composition of the fruit peel blends is reported in Table 4. Magnesium (Mg) levels varied significantly across the samples, with PCGA recording the highest concentration (34.8 ± 0.02 mg/100 g), while WGRC had the lowest (24.7 ± 0.03 mg/100 g). Potassium (K) ranged from 156.3 ± 0.02 mg/100 g (WGRC) to 204.6 ± 0.03 mg/100 g (OLMA), with all the values being significantly different (*p* < 0.05). Sodium (Na) was also found in significant quantities across the nine samples. OLMB had the highest content of calcium (60.2 ± 0.01 mg/100 g), significantly differing from the others, while WGRC recorded the lowest calcium concentration (40.3 ± 0.01 mg/100 g).

**TABLE 4:** Macro element content of the fruit peel blends (mg/100 g).

Samples	Magnesium	Potassium	Sodium	Calcium
OLMA	30.4 ± 0.01^e^	204.6 ± 0.03^i^	21.4 ± 0.01^d^	52.4 ± 0.02^f^
OLMB	32.9 ± 0.01^g^	194.6 ± 0.03^h^	23.5 ± 0.03^f^	60.2 ± 0.01^h^
OLMC	28.6 ± 0.03^d^	164.8 ± 0.01^e^	19.6 ± 0.03^a^	48.4 ± 0.02^e^
PCGA	34.8 ± 0.02^h^	172.4 ± 0.02^f^	24.8 ± 0.01^i^	56.4 ± 0.01^g^
PCGB	24.9 ± 0.02^b^	163.1 ± 0.01^b^	21.6 ± 0.03^e^	48.4 ± 0.01^d^
PCGC	31.6 ± 0.03^f^	178.3 ± 0.02^g^	20.8 ± 0.01^c^	45.3 ± 0.01^b^
WGRA	25.4 ± 0.01^c^	163.8 ± 0.05^c^	23.6 ± 0.03^g^	52.4 ± 0.02^f^
WGRB	24.9 ± 0.02^b^	164.5 ± 0.03^d^	24.6 ± 0.03^h^	46.2 ± 0.01^c^
WGRC	24.7 ± 0.03^a^	156.3 ± 0.02^a^	20.6 ± 0.01^b^	40.3 ± 0.01^a^

*Note:* Values in the same column with different superscripts are significantly different at *p* < 0.05. Values are expressed as mean ± SEM, *n* = 2.

#### 3.2.2 Microelement Content of the Fruit Peel Blends

Table 5 presents the microelement composition (Manganese, Zinc, Iron, Selenium, and Copper) of the nine fruit peel blends. Manganese (Mn) levels varied slightly across the samples, with OLMA, OLMC, and WGRC recording the same and the lowest value (0.9 mg/100 g), while PCGC had the highest Mn concentration (1.2 ± 0.00 mg/100 g). Zinc (Zn) content was highest in OLMB, WGRA, and WGRC (1.3 ± 0.01 mg/100 g), significantly (*p* < 0.05) differing from WGRB, which had the lowest concentration (0.8 ± 0.01 mg/100 g). Iron (Fe) levels were highest in OLMA (2.2 ± 0.00 mg/100 g), significantly exceeding all other samples, while OLMB and OLMC recorded the lowest Fe content (0.9 ± 0.01 mg/100 g). Selenium (Se) had the lowest values, which ranged from 0.19 ± 0.01 mg/100 g in OLMC and WGRC to 0.19 ± 0.01 mg/100 g in PCGB. Almost all the fruit peel blends had the same concentration of Copper (Cu) i.e., 0.7 ± 0.01 mg/100 g, except for two samples—OLMA and OLMB which recorded a slightly lower value of 0.6 ± 0.01 mg/100 g which still differed significantly (*p* < 0.05) from the other values.

**TABLE 5:** Microelement content of the fruit peel blends (mg/100 g).

Samples	Mn (mg/100 g)	Zn (mg/100 g)	Fe (mg/100 g)	Se (mg/100 g)	Cu (mg/100 g)
OLMA	0.9 ± 0.00^a^	1.1 ± 0.02^d^	2.2 ± 0.00^g^	0.10 ± 0.00^b^	0.6 ± 0.01^a^
OLMB	1.0 ± 0.01^b^	1.3 ± 0.01^g^	0.9 ± 0.01^a^	0.13 ± 0.00^c^	0.6 ± 0.01^a^
OLMC	0.9 ± 0.00^a^	1.0 ± 0.00^b^	0.9 ± 0.01^a^	0.09 ± 0.00^a^	0.7 ± 0.00^b^
PCGA	1.0 ± 0.00^b^	1.2 ± 0.00^e^	1.2 ± 0.01^d^	0.12 ± 0.00^c^	0.7 ± 0.00^a^
PCGB	1.1 ± 0.01^d^	1.1 ± 0.00^c^	1.2 ± 0.03^e^	0.19 ± 0.01^d^	0.7 ± 0.01^a^
PCGC	1.2 ± 0.00^e^	1.2 ± 0.01^e^	1.3 ± 0.01^f^	0.13 ± 0.00^c^	0.7 ± 0.00^b^
WGRA	1.0 ± 0.01^c^	1.3 ± 0.01^f^	1.0 ± 0.01^b^	0.14 ± 0.00^b^	0.7 ± 0.01^b^
WGRB	1.0 ± 0.02^c^	0.8 ± 0.01^a^	1.1 ± 0.02^c^	0.11 ± 0.00^b^	0.7 ± 0.01^a^
WGRC	0.9 ± 0.03^a^	1.3 ± 0.01^f^	1.0 ± 0.01^b^	0.09 ± 0.00^a^	0.7 ± 0.01^a^

*Note:* Values in the same column with different superscripts are significantly different at *p* < 0.05. Values are expressed as mean ± SEM, *n* = 2.

### 3.3 Antioxidant Activity of the Fruit Peel Blends

**FIGURE 2:**
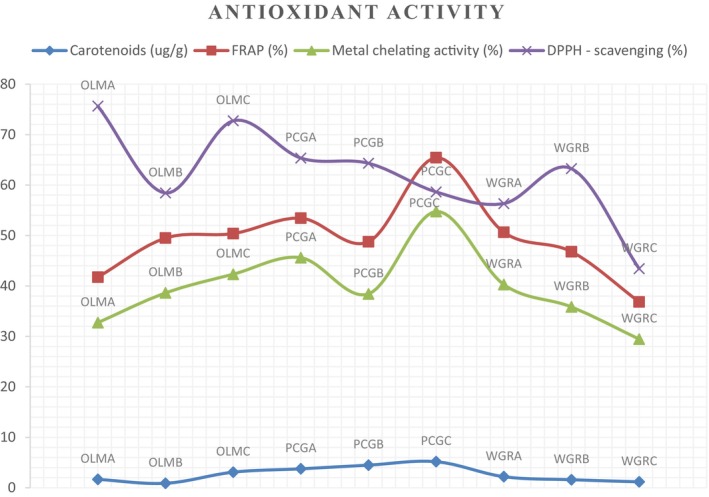
Antioxidant activity of the fruit peel blends.

The four parameters analyzed for antioxidant activity of the nine samples are reported in Figure 2; they are carotenoids, FRAP, metal chelating activity, and DPPH scavenging. Generally, significant differences (*p* < 0.05) were observed among the nine samples, with PCGC consistently exhibiting the highest values in most antioxidant parameters, except in the DPPH scavenging activity, while WGRC had the lowest antioxidant activity across the measured indices (except in carotenoids, where it came 2nd to the least). PCGC recorded the highest concentration of carotenoids (5.2 ± 0.04 μg/g), which was significantly (*p* < 0.05) higher than all other samples; OLMB had the lowest carotenoids (0.9 ± 0.01 μg/g). For FRAP, the values ranged from as low as 36.8% ± 0.06% in WGRC up to 65.4% ± 0.03% in PCGC. The metal chelating activity was found to be significantly higher in PCGC (54.8% ± 0.04%) compared to other samples, with WGRC exhibiting the lowest value (29.5% ± 0.03%). DPPH scavenging activity was notably highest in OLMA (75.7% ± 0.04%), which also significantly differs from other samples, with WGRC recording the least DPPH scavenging activity (43.4% ± 0.03%).

### 3.4 Phytochemical Composition of the Fruit Peel Blends

The phytochemical composition (Tannin, Oxalate, Phytate, Phenol, Flavonoid, HCN, and TPC) of nine samples are presented in Table 6. Tannin content varied significantly, with PCGA (2.2 ± 0.03 mg/100 g) exhibiting the highest concentration, while WGRC had the lowest (0.9 ± 0.01 mg/100 g). Also, oxalate was highest in PCGA (5.4 ± 0.03 mg/100 g), whereas WGRC recorded the lowest concentration (2.6 ± 0.04 mg/100 g). Phytate values varied between 0.9 ± 0.01 mg/100 g (WGRA) and 1.6 ± 0.03 mg/100 g (PCGA), showing significant differences (*p* < 0.05) among the samples. Flavonoids were the most predominant among the phytochemicals analyzed, with OLMB recording significantly higher value (76.8 ± 0.04 mg/100 g) while PCGC had the least (42.8 ± 0.01 mg/100 g). On the other hand, HCN showed the lowest phytochemical concentrations, with none of the samples having up to 2.0 mg/100 g. PCGA had the highest total phenolic content (10.4 ± 0.04 mg/100 g), significantly (*p* < 0.05) surpassing the others, while WGRA and WGRC had the lowest TPC (5.9 ± 0.02 mg/100 g each).

**TABLE 6:** Phytochemical composition of the fruit peel blends (in mg/100 g).

Samples	Tannin	Oxalate	Phytate	Phenol	Flavonoids	HCN	TPC
OLMA	1.4 ± 0.03^d^	4.0 ± 0.02^e^	1.2 ± 0.01^e^	4.0 ± 0.03^e^	63.5 ± 0.04^f^	0.8 ± 0.04^b^	7.4 ± 0.02^d^
OLMB	1.7 ± 0.01^e^	4.7 ± 0.01^g^	1.0 ± 0.04^c^	4.8 ± 0.04^f^	76.8 ± 0.04^i^	1.3 ± 0.03^f^	7.9 ± 0.01^e^
OLMC	1.6 ± 0.04^e^	4.9 ± 0.06^g^	1.0 ± 0.01^d^	5.0 ± 0.06^g^	57.6 ± 0.03^d^	1.3 ± 0.01^e^	8.6 ± 0.07^f^
PCGA	2.2 ± 0.03^f^	5.4 ± 0.03^i^	1.6 ± 0.03^h^	5.4 ± 0.03^h^	61.4 ± 0.01^e^	1.0 ± 0.01^c^	10.4 ± 0.04^g^
PCGB	1.6 ± 0.01^e^	4.6 ± 0.04^f^	1.2 ± 0.03^f^	7.4 ± 0.01^i^	50.4 ± 0.01^b^	1.7 ± 0.01^g^	7.5 ± 0.02^d^
PCGC	1.3 ± 0.02^c^	3.9 ± 0.01^d^	0.98 ± 0.01^b^	3.9 ± 0.03^d^	42.8 ± 0.01^a^	1.8 ± 0.01^h^	6.9 ± 0.02^c^
WGRA	1.2 ± 0.01^b^	3.4 ± 0.03^b^	0.9 ± 0.01^a^	3.4 ± 0.03^b^	75.5 ± 0.09^h^	1.1 ± 0.02^d^	5.9 ± 0.02^a^
WGRB	1.2 ± 0.01^b^	3.6 ± 0.03^c^	1.1 ± 0.01^c^	3.6 ± 0.03^c^	72.8 ± 0.06^g^	1.9 ± 0.01^i^	6.3 ± 0.01^b^
WGRC	0.9 ± 0.01^a^	2.6 ± 0.04^a^	1.4 ± 0.01^g^	2.6 ± 0.04^a^	56.4 ± 0.01^c^	0.6 ± 0.01^a^	5.9 ± 0.02^a^

*Note:* Values in the same column with different superscripts are significantly different at *p* < 0.05. Values are expressed as mean ± SEM, *n* = 2.

## 4 Discussion

The analytical results of the nine fruit peel blends show that they all possess essential micronutrients, antioxidants, and phytochemicals in significant and useful quantities that can definitely impact positively on the nutritional status of consumers. The findings of this study confirm the reports of similar studies which analyzed some of the fruit peels used in this study to formulate the blends. Naseem et al. (2012), Feumba et al. (2016) and Onyenweaku and Kesa (2024) also report significant micronutrient and antioxidant properties of the studied fruit peels. Micronutrients—both minerals and vitamins play—crucial roles in the maintenance of health and prevention of diseases. Some of these micronutrients, like selenium, zinc, vitamins C and E, also possess very good antioxidant properties. Ismail et al. (2014) report that fruits as well as their various parts (such as seeds, peels, pulp) are good sources of dietary minerals. Considering the recent rise in malnutrition statistics, it has become necessary for researchers to begin to come up with sustainable solutions to the global burden of disease—both hidden hunger and diet‐related non‐communicable diseases (NCDs).

Incorporating more natural, organic plant‐based foods into meals has proven to be an effective strategy for reducing the burden of diseases that have become more prevalent with industrialization/urbanization. Rather than discard these useful fruit peels (constituting food waste), a lot can be done with them which will not only increase the nutritional value of foods but also strengthen the body's immune system; this includes making fruit sprinkles, processing them as blends to be used as food additives (as done in this study), and even incorporating them into food systems like baked products and smoothies (Onyenweaku et al. 2024). Comparing the results of this study with those from the preliminary phase reveals that the mineral content remains largely similar, except for potassium, which is significantly higher in the blends.

The decision to formulate fruit peels into blends was based on the understanding that different peels possess unique flavors and can also complement the nutrient profile of other peels. The findings of this study align with previous research by Hussain et al. (2023), which highlighted the high potassium content in banana peels, as well as significant quantities of iron and magnesium in apple peels. Alfonzo et al. (2006) highlight that increased potassium availability may be pivotal in managing respiratory distress, renal disorders, and cardiac dysfunction. Moreover, the iron content in these blends is impressive, providing up to 25% of the recommended daily iron intake for men (8 mg) and substantially contributing to the 18 mg daily requirement for women (Otten et al. 2006). Incorporating these dietary additives could significantly improve the iron status of children in areas where iron deficiency anemia (IDA) is common. In low‐income countries, where many cannot afford animal‐source foods like meat, fish, and eggs—key sources of dietary iron—these fruit peel blends offer a more affordable and accessible alternative.

For the vitamins, it is note worthy that the PCG blends had significantly higher concentrations of the six vitamins that were analyzed, especially beta‐carotene and vitamin C. The PCG blend, consisting of pineapple, cucumber, and green apples, is made from tropical fruits that are readily available year‐round. It is advisable that these fruit peels be explored for their potential vitamin content as well. Notably, beta‐carotene, a precursor to vitamin A, plays a vital role in maintaining healthy eyes and skin while also serving as a powerful antioxidant that helps prevent chronic diseases (Bohn et al. 2019). Furthermore, the ferric reducing power assay provides a clear ranking of the antioxidant capacity of the fruit peel blends tested. In a very recent and similar study carried out in India by Sharma et al. (2025), orange and sweet lemon peels exhibited the highest antioxidant activity, closely followed by pomegranate, while banana showed relatively lower activity. These results suggest that citrus fruit peels (orange, lemon, mandarin) are particularly rich in antioxidants, supporting their potential use in health applications, such as preventing oxidative damage, managing chronic diseases, and even in food preservation. This finding is consistent with studies by Ashraf et al. (2024) and Park et al. (2012), which attributed high antioxidant activity in orange peels to their abundant phenolic compounds and flavonoids. Additionally, DPPH scavenging activity was the most prominent antioxidant property observed; the citrus blends (OLM) excelled in DPPH activity, whereas the PCG blend recorded the highest values for the other antioxidant parameters, and the citrus blends (OLM) had the highest DPPH scavenging activity while the PCG blend recorded the highest values for all the other antioxidant parameters. Notably, the PCGC sample was particularly higher than all the others in the antioxidant parameters except DPPH scavenging. This PCG result can be explained by their significantly higher carotenoid and TPC, just as the high carotenoid content of the OLM blends may be a result of the mandarin peel in those formulations. Similarly, Suleria et al. (2020) offer evidence supporting the use of phenolic‐rich fruit peels (most of which were also analyzed in this study) as valuable ingredients in food, feed, and nutraceutical formulations.

The fruit peel blends contain antinutrients—such as oxalate, phytate, and HCN—but their levels remain within safe limits recommended by the WHO (50–100 mg/day) (Gibson et al. 2018). In fact, one would need to consume an unusually large amount of these peels in a single day to exceed these limits, so there is minimal cause for concern. The zinc‐phytate molar ratio in most of the blends is about 1:1, which is slightly above the European Food Safety Authority's dietary threshold of 4:1, since high phytate levels can inhibit iron and zinc absorption (UNICEF 2021). Traditional processing techniques like fermentation, soaking, sprouting, and phytase hydrolysis can reduce phytate concentrations, further improving the bioavailability of these essential minerals (Gibson et al. 2018; Popova and Mihaylova 2019). Recent research, however, has questioned the overall impact of these compounds, termed “antinutrients.” While some studies suggest that compounds including lectins, oxalates, phytates, phytoestrogens, and tannins may reduce nutrient bioavailability, other research indicates they might also offer health benefits (Petroski and Minich 2020; Gautam et al. 2020). Ultimately, both the concentration of these compounds and the food matrix in which they are found are key to determining their nutritional impact. Summarily, fruit processing by‐products can serve as valuable sources of bioactive substances, which have demonstrated promising applications in the food, cosmetics, and pharmaceutical industries. Effective ways of optimizing their maximizing their benefits should be explored in order to enhance sustainability and add economic value to fruit waste management.

## 5 Conclusion

Addressing food insecurity remains a pressing global concern, exacerbated by the rising prevalence of malnutrition and NCDs. Limited access to nutritious diets, coupled with unhealthy lifestyle choices, has contributed to these challenges, hence the current shift from animal foods to plant‐based diets. Fruits have well‐known and proven health benefits, yet their peels, which are often discarded, contain valuable micronutrients, antioxidants, and phytochemicals. This study demonstrated that formulating fruit peels into blends as sprinkles for incorporation into diets, drinks, and even baked products can go a long way to enhance their nutritional profile, providing essential minerals, vitamins, and antioxidants. The bioactive compounds identified in significant concentrations within these fruit peel blends have been reported to exhibit anti‐inflammatory, antimicrobial, and potential anticancer properties. These research findings highlight the need for further investigation into the potential applications of these bioactive compounds and the development of effective methods to preserve their stability during storage. Therefore, utilizing fruit peels as food additives presents a sustainable approach to minimizing food waste in households and the food industry at large while improving the nutritional status of consumers.

## Author Contributions

**Eridiong Onyenweaku:** conceptualization (lead), investigation (equal), resources (equal), writing – original draft (lead). **Hema Kesa:** formal analysis (lead), project administration (lead), supervision (equal), writing – review and editing (equal). **Patricia Ebai:** data curation (lead), investigation (equal), validation (lead), writing – review and editing (equal).

## Ethics Statement

This study does not involve any human or animal testing.

## Consent

The authors have nothing to report.

## Conflicts of Interest

The authors declare no conflicts of interest.

## Data Availability

The data that support the findings of this study are available from the corresponding author upon request.

## Appendix 1 Vitamin Analyses

### Determination of Vitamin A Content

The AOAC (2010) colorimetric method was used to measure vitamin A by detecting its reaction with SbL_3_ at 620 nm. Pyrogallol was added to a 2 g sample before saponification with 200 mL alcoholic KOH in a water bath for 30 min. The solution was extracted with 2.5 mL hexane and washed with an equal volume of water. The extract was filtered through filter paper containing 5 g anhydrous Na_2_SO_4_. After evaporating hexane, 1 mL chloroform and SbL_3_ were added to extract the blank. Absorbance readings were taken using a colorimeter set to zero absorbance or 100%.

Calculation:

$$\text{Vitamin}\;A=A_{62\text{0nm}}\times \mathrm{SL}\times \left(V/\mathrm{Wt}\right)\;\mathrm{mg}$$

where, *A*_620nm_ = absorbance at 620 nm, SL = Slope of standard curve (Vit. A conc.) + *A*_620nm_ reading, *V* = Final volume in colorimeter tube, Wt = weight of sample.

### Determination of Vitamin B_1_ Content

Thiamine (B_1_) content was determined using the scalar analyzer method of AOAC (2010); 5 g of each sample was homogenized in 5 mL normal ethanoic sodium hydroxide solution. The homogenate was filtered and made up to 100 mL with the extract solution. A 10 mL aliquot of the extract was dispensed into a flask and 10 mL of potassium dichromate solution added. The resultant solution was incubated for 15 min at room temperature (25°C ± 1°C). The absorption was read from the spectrophotometer at 360 nm using a reagent blank to standardize the instrument at zero.

Calculation:

$$\text{Thiamine}\;\left(\mathrm{mg}/100\;g\right)=\frac{100}{W}\times \frac{a_{u}}{a_{s}}\times c\times d$$

where, *W* = weight of sample analyzed, *a*_u_ = absorbance of the sample solution, *a*_s_ = absorbance of standard solution, *c* = concentration of standard solution, *d* = dilution factor.

### Determination of Vitamin B_2_ Content

Riboflavin (B_2_) was determined according to AOAC (2010) fluorimetric method; 2 g of sample was placed in a conical flask and 50 mL of 0.2 N HCl was added to the sample, boiled for 1 h, and then cooled. The pH was adjusted to 6.0 using sodium hydroxide; 1 N HCl was added to the sample solution to lower the pH to 4.5. The solution was filtered into 100 mL measuring flask and made to volume with water. To eliminate interference, two test tubes were prepared. Tube 2 contained 10 mL filtrate and 1 mL riboflavin standard. Both tubes received 1 mL glacial acetic acid, 0.5 mL KMnO_4_, and 0.5 mL H_2_SO_4_. Fluorescence was measured at 470 nm excitation and 525 nm emission. Sodium hydrogen sulfate was added, and a blank reading was recorded within 10 s.

Calculation:

$$\text{Riboflavin}\;\mathrm{mg}/g=\frac{X}{Y-x}\times \frac{1}{w}$$

where, *W* = weight of sample, *X* = reading of sample—blank reading, *Y* = reading of sample + standard tube (2)—reading of sample + standard blank.

### Determination of Vitamin B_3_ Content

The spectrophotometric method described by Okwu and Ndu (2006) was employed for niacin (B_3_). A measured weight sample of 5 g was mixed with 30 mL of normal H_2_SO_4_ solution and shaken for 30 min, then filtered to obtain the extract. The extract was alkalized with NH_4_OH, then 10 mL was treated with 5 mL potassium ferrocyanide and 5 mL 0.02N H_2_SO_4_. After 5 min, absorbance was measured at 470 nm. A standard niacin solution underwent the same process, and both absorbances were recorded using a spectrophotometer.

Calculation:

$$\text{Niacin}\;\left(\mathrm{mg}/100\;g\right)=\frac{100}{W}\times \frac{A_{u}}{A_{s}}\times c\times \frac{V_{f}}{V_{a}}$$

*W* = weight of the sample analyzed, *A*_u_ = absorbance of sample, *A*_s_ = absorbance of standard, *C* = concentration (mg/mL) of standard niacin solution, *V*_f_ = total volume of filtrate (extract), *V*_a_ = volume of extract analyzed.

### Determination of Vitamin C Content

Ascorbic acid was determined using the titrimetric method described by AOAC (2010); 5 g of the sample was dispensed in 50 mL of EDTA/TCA solution and homogenized. The homogenate was filtered with Whatman No. 42 filter paper, and the extractant was used to wash the residue until 50 mL filtrate was obtained. A 20 mL portion of the filtrate was measured into a conical flask, and 10 mL of 30% potassium solution was added to it, mixed well, and then followed by 1% starch solution. The mixture was titrated against 0.01 M CuSO_4_ solutions. A reagent blank was also titrated.

The vitamin C content was calculated based on the relationship that 1 mL 0.01 M CuSO_4_ = 0.88 mg vitamin C.

$$\text{Vitamin}\;C\;\left(\mathrm{mg}/100\;g\right)=\frac{100}{w}\times 0.88\times \left(T-B\right)\times \frac{V_{f}}{V_{a}}$$

*w* = weight of sample, *T* = Titer value of sample, *B* = Titer valve of blank, *V*_t_ = total extract volume, *V*_a_ = volume of extract titrated.

### Determination of Vitamin E Content

Vitamin E was determined using the method described by AOAC (2010). One gram of sample was weighed into a 100 mL flask; 10 mL of absolute alcohol and 20 mL of alcoholic tetraoxosulphate (VI) acid (H_2_SO_4_) were added. Ten milliliters of the clear solution was pipetted into a test tube and heated in a water bath at 90°C for 3 min. This was allowed to cool. The absorbance was read in a spectrophotometer at 470 nm wavelength.

Calculation:

$$\text{Vitamin}\;E\;\text{in}\;\mathrm{mg}/100\;g=\frac{a-b\times c\;}{s-b\times w}$$

where, *a* = absorbance of test sample, *b* = absorbance of the standard solution, *c* = concentration of standard in mg/100 g; *w* = weight of the sample used.

## Appendix 2 Phytochemical Analysis

### Determination of Tannin Content

Tannin content was determined using the method described by AOAC (2005). Five‐gram sample was dispersed in 50 mL of distilled water and shaken. The mixture was allowed to stand for 30 min at 28°C before it was filtered through Whatman No. 42 grade of filter paper. Two milliliters of the extract were dispersed into a 50 mL volumetric flask. Similarly, 2 mL standard tannin solution (tannic acid) and 2 mL of distilled water were put in separate volumetric flasks to serve as standard and reagent was added to each of the flask and then 2.5 mL of saturated Na_2_CO_3_ solution was added. The content of each flask was made up to 50 mL with distilled water and allowed to incubate at 28°C for 90 min. Their respective absorbance was measured in a spectrophotometer at 260 nm using the reagent blank to calibrate the instrument at zero.

### Determination of Oxalate Content

Oxalate was determined titrimetrically using the method of AOAC (2020). Five (5) grams of the sample was weighed into a 100 mL beaker, 20 mL of 0.30 NHCl was added and warmed on magnetic plate and stirred for 1 h. The extract was filtered into a 100 mL volumetric flask and diluted to the 100 mL mark of the flask. Five (5) milliliter extract was pipette into a conical flask and made alkaline with 1.0 mL of 5 N ammonium hydroxide. Indicator paper was placed in the conical flask to show the alkaline regions. Two (2) drops of phenolphthalein indicator was added, 1 mL glacial acetic acid was added (excess acid decolorized the solution). One milliliter of 5% CaCI_2_ was then added and the mixture was allowed to stand for 3 h after which it was centrifuged at 3000 rpm for 15 min. The supernatants were discarded and the precipitates, washed three times with hot water with thorough mixing and centrifuging each time. Two milliliters of 3NH_2_SO_4_ was added to each tube and the precipitates dissolved by warming in a water bath (70°C–80°C). The content of the tubes was carefully poured into a clean conical flask and titrated with freshly prepared 0.05 M KMnO_4_ at room temperature until the first pink color appeared and till the solution became colorless. The solution was then warmed to 70°C–80°C and titrated until a permanent pink color that persisted for at least 30 s was attained. The oxalate content was calculated as sodium oxalate equivalent.

### Determination of Phytate Content

Phytate content was determined using a spectrophotometric method as described by AOAC (2005). Half a gram (0.5 g) of sample was weighed into a 500 mL flat‐bottom flask. The flask was placed in a shaker, and the sample was extracted with 2.4% HCl for 1 h. The aliquot was filtered, and 5 mL of the filtrate was pipetted and diluted to 25 mL with distilled water. Fifteen milliliters of NaCl was added to 10 mL of the diluted sample, and this was passed through an amplet resin (200–400 mesh) to elude inorganic phosphorus. About 15 mL of 0.7 M sodium chloride (NaCl) was added to the solution, which was mixed on a vortex mixer for 5 s. The mixture was then centrifuged for 10 min, and the supernatant was read at 520 nm wavelength in a UV spectrophotometer. The phytate concentration was read off from a standard curve prepared with standard inositol phytate, and the value was expressed in mg/l00g using the formula:

$$\begin{array}{cc} \text{Phytate}\;\left(\mathrm{mg}\right)=\; & \frac{\text{conc}.\;\text{of phytate}\;\left(\mathrm{mg}/100\;g\right)\;\text{from standard curve}}{\text{Weight of sample}}\\ & \times \text{dilution factor} \end{array}$$

### Determination of Flavonoid

The total flavonoid content of the extract was determined using a slightly modified method reported by Meda et al. (2005). About 0.5 mL of the sample extract was mixed with 0.5 mL method, 50 μL 10% AlCl_3_, 50 μL I M potassium acetate and 1.4 mL water and allowed to incubate at room temperature for 30 min. The absorbance of the reaction mixture was subsequently measured at 415 nm and the total flavonoid content calculated as quercetin equivalent.

### Determination of Hydrogen Cyanide (HCN) Content

This was determined by the alkaline picrate colourimetric method by AOAC (2005). One gram of each sample was weighed into a conical flask and 200 mL of distilled water added to it. Each sample was thoroughly mixed. A strip of alkaline picrate paper was suspended over the mixture, with the aid of a rubber stopper, in such a way that the paper did not touch the surface of the mixture. The set up was incubated for 18 h at room temperature. At the end of the incubation period, the picrate paper was carefully removed and placed in 60 mL of distilled water. Meanwhile a standard cyanide solution was prepared and treated as described above. The absorbance of the standard and the sample were measured in a spectrophotometer at 540 nm. The cyanide content (HCN) in mg/kg was calculated using the formula:

$$\mathrm{HCN}\;\left(\mathrm{mg}/\mathrm{kg}\right)=\left(100/W\right)\times A_{u}\;\left(A_{s}\right)$$

where, *W* = weight of sample analyzed (g), *A*_u_ = Absorbance of sample (nm), *A*_s_ = Absorbance of the standard HCN solution (nm).

### Determination of Total Phenolic Content (TPC)

The total phenol content was determined according to the method of Meda et al. (2005) using gallic acid as standard. The sample extract (0.5 mL) was mixed with 25 mL Folin‐ciocalteau reagent (v/v) (previously diluted 10‐fold with distilled water) and 2 mL sodium bicarbonate (7.5 % w/v) and the mixture was diluted to 100 mL with distilled water. The solution was incubated for 40 min at 45°C for color development; the absorbance was measured at 765 nm. Total phenolic content was expressed as gallic acid equivalents (the concentration of gallic acid was established from a calibration curve) in mg per g fresh weight (mg GAE/g).
